# Mesenchymal stem cells overexpressing hepatocyte nuclear factor-4 alpha alleviate liver injury by modulating anti-inflammatory functions in mice

**DOI:** 10.1186/s13287-019-1260-7

**Published:** 2019-05-27

**Authors:** Zhenxiong Ye, Wenfeng Lu, Lei Liang, Min Tang, Yunfeng Wang, Zhen Li, Heping Zeng, Aili Wang, Moubin Lin, Lei Huang, Hui Wang, Hai Hu

**Affiliations:** 1 Department of General Surgery, Yangpu Hospital, Tongji University School of Medicine, 450 Teng Yue Road, Shanghai, 200090 China; 2 Department of Hepatobiliary Surgery, Eastern Hepatobiliary Surgery Hospital, The Second Military Medical University, Shanghai, China; 3 Center of Gallbladder Disease, Shanghai East Hospital, Tongji University School of Medicine, 150 Ji Mo Road, Shanghai, 201200 China

**Keywords:** Mesenchymal stem cells, Hepatocyte nuclear factor-4 alpha, Liver injury, Immune regulation

## Abstract

**Background:**

Mesenchymal stem cells (MSCs) can migrate to tissue injury sites where they can induce multipotential differentiation and anti-inflammation effects to treat tissue injury. When traditional therapeutic methods do not work, MSCs are considered to be one of the best candidates for cell therapy. MSCs have been used for treating several injury- and inflammation-associated diseases, including liver cirrhosis. However, the therapeutic effect of MSCs is limited. In some cases, the anti-inflammatory function of naïve MSCs is not enough to rescue tissue injury.

**Methods:**

Carbon tetrachloride (CCl_4_) was used to establish a mouse liver cirrhosis model. Enhanced green fluorescence protein (EGFP) and hepatocyte nuclear factor-4α (HNF-4α) overexpression adenoviruses were used to modify MSCs. Three weeks after liver injury induction, mice were injected with bone marrow MSCs via their tail vein. The mice were then sacrificed 3 weeks after MSC injection. Liver injury was evaluated by measuring glutamic-pyruvic transaminase (ALT) and glutamic oxalacetic transaminase (AST) levels. Histological and molecular evaluations were performed to study the mechanisms.

**Results:**

We found that HNF-4α-overexpressing MSCs had a better treatment effect than unmodified MSCs on liver cirrhosis. In the CCl_4_-induced mouse liver injury model, we found that HNF-4α-MSCs reduced inflammation in the liver and alleviated liver injury. In addition, we found that HNF-4α promoted the anti-inflammatory effect of MSCs by enhancing nitric oxide synthase (iNOS) expression, which was dependent on the nuclear factor kappa B (NF-κB) signalling pathway.

**Conclusions:**

MSCs overexpressing HNF-4α exerted good therapeutic effects against mouse liver cirrhosis due to an enhanced anti-inflammatory effect. Gene modification is likely a promising method for improving the effects of cell therapy.

**Electronic supplementary material:**

The online version of this article (10.1186/s13287-019-1260-7) contains supplementary material, which is available to authorized users.

## Background

Liver cirrhosis is the end stage of chronic liver injury and is characterized by liver structure disorder, nodule formation and liver function loss, most likely resulting in liver failure or hepatocellular carcinoma (HCC) [1–3]. Currently, orthotopic liver transplantation (OLT) is considered to be the most effective treatment for liver cirrhosis; however, it is limited by donor shortages, immunological rejection and expensive medical costs [4].

In recent years, stem cell transplantation has become a promising alternative option for most diseases. MSCs have been reported to be an effective treatment for liver injury [5] because they are easy to isolate, proliferate well in vitro and have low immunogenicity and no ethical issues [6]. MSCs have been shown to be able to migrate to injury sites and exert anti-inflammatory or differentiation effects. MSCs have been shown to be highly effective in treating tissue injury and degenerative diseases, such as myocardial infarction, spinal cord injury, bone damage, cornea damage, liver cirrhosis and other tissue injuries [6]. However, there are some issues limiting the use of MSCs [7]. It has been reported that 1–5 × 10^6^ cells/kg are needed to treat patients [8, 9]. As a result, MSCs need to be expanded beyond passage 5 to generate sufficient numbers for clinical treatment. However, reports have demonstrated that adult BM-MSCs lose their immunosuppressive capabilities in early passages (5–7) [10]. In addition, MSCs from different sources have been shown to differentially regulate the immune response [11]. Thus, naïve MSCs are not enough to rescue injury in some cases. However, there are some things that can be done to promote the anti-inflammatory effects of MSCs.

HNF-4α is a nuclear transcription factor that can initiate the expression of a series of mature liver cell markers and plays an important role in liver cell maturation [12, 13]. Studies have reported that HNF-4α overexpression via adenovirus can reduce liver damage and treat liver cancer [14], but both lentiviruses and adenoviruses have certain risks for direct application in vivo, such as tumourigenesis and strong inflammatory responses.

In the present study, to eliminate the risks of virus application in vivo and to enhance the treatment of MSCs, we used an adenovirus as a vector for overexpressing HNF-4α in MSCs. We transplanted bone marrow MSCs overexpressing HNF-4α (HNF-4α-MSCs) into mice with chronic liver injury caused by CCl_4_. An adenovirus overexpressing EGFP was used as a control. To our surprise, the therapeutic effect of MSCs on liver injury after transfection with the HNF-4α-adenovirus was highly improved. However, the mechanism requires further study. We focused on changes in hepatocyte differentiation and inflammation regulation. Surprisingly, we found that hepatocyte differentiation induction via HNF-4α in vivo was not the key reason why MSCs reduced liver injury. Increased immune regulation by MSCs was found to play a dominant role in liver injury repair. Finally, we demonstrated that HNF-4α could facilitate the anti-inflammatory function of MSCs by increasing the protein expression of inducible nitric oxide synthase (iNOS) by activating the NF-κB signalling pathway.

## Materials and methods

### Animals and experimental design

Male C57BL/6 mice (6–8 weeks old, weighing 20–25 g) were purchased from the Shanghai Laboratory Animal Center and housed in pathogen-free conditions with a 12/12 h light/dark illumination cycle and free access to food and water. Animal protocols were approved by the TongJi University Animal Care Committee, and all animal experiments conformed to the relevant regulatory standards. CCl_4_ is commonly used for mouse liver injury models. Mice were separated into four groups: control group (olive oil was administered via intragastric gavage), cirrhosis group (20% CCl_4_ was used at a concentration of 5 mL/kg twice a week), cirrhosis + EGFP-MSC group (1 × 10^6^ EGFP-MSCs were injected via tail vein at the third week) and cirrhosis + HNF-4α-MSC group (1 × 10^6^ HNF-4α-MSCs were injected via tail vein at the third week). MSCs at passage 4 were transfected with adenovirus and then injected into the mice. To assess the effect of cell therapy, the mice were sacrificed 3 weeks after injection.

### Isolation and culture of primary MSCs

Bone marrow MSCs were isolated from male C57BL/6 mice according to Heng Zhu’s protocol [15]. Bone marrow was acquired by flushing the femurs and tibias of mice with culture medium. Then, the cells were blown into a single cell suspension and seeded into a 10-cm dish with 20 mL medium. Five days later, the medium was changed, and the cells were passaged when confluent. The cells were considered MSCs and used for experiments after 3 passages. MSCs were identified by testing their ability to differentiate to adipocytes and osteocytes.

### Recombinant adenovirus

A recombinant HNF-4α overexpression adenovirus and control adenovirus were purchased from Genechem Biotech, Inc. Shanghai, China. MSC infection (passage 4) was performed according to the manufacturer’s protocol.

### Histological and immunohistochemical analyses

Mouse liver tissues were fixed in formalin for 24 h and embedded in paraffin. Five-micron-thick sections were made from paraffin-embedded liver tissues and then stained with haematoxylin and eosin to detect histological structure and with Sirius red to detect liver fibrosis. For immunohistochemical staining, the sections were incubated with primary antibodies to ALB (ab192603, 1:300, Abcam) and CD68 (ab955, 1:200, Abcam) according to previously described protocols [16].

### Biochemical analysis

Mouse blood was collected when the mice were sacrificed. The samples were kept at 4 °C overnight and then centrifuged at 3000 rpm for 10 min for serum separation. To measure liver injury, the serum samples were analysed for glutamic-pyruvic transaminase and glutamic oxalacetic transaminase levels by a biochemical analyser (DRI-CHEM 55500 V, Fuji Medical System, Japan).

### Transwell assay

Ten thousand EGFP-MSCs and HNF-4α-MSCs (both passage 4) were plated into the upper chamber of a 24-well plate. Culture medium with 5% FBS was added to the lower chamber, while serum-free medium was added into the upper chamber. After 72 h, the migrated cells were stained with crystal violet and counted.

### CCK-8 assay

A total of 3000 MSCs, EGFP-MSCs and HNF-4α-MSCs (all passage 4) were plated into a 96-well plate; 6 replicates per group were used for this experiment. After 48 h, CCK-8 solution was added to the plate at a ratio of 100 μL fresh serum-free culture medium:10 μL CCK-8 solution, and the cells were incubated for 1 h in a 37 °C incubator. Then, the OD values were detected at a wavelength of 450 nm.

### MSC co-culture with A1.1 cells

MSCs (passage 4) were treated with 10 ng/mL IFN-γ and 10 ng/mL TNF-α for 12 h and then co-cultured with A1.1 cells at a ratio of 1 MSC:10 A1.1 cells for 24 h. Then, the proliferation of A1.1 cells was measured by ^3^H-Tdr assay.

### ^3^H-Tdr assay

A total of 0.5 μCi of ^3^H-thymidine was added to each well of a 96-well plate 6 h before the cultures were terminated by freezing. The cells were harvested after the plates were thawed, and incorporated ^3^H-Tdr was assessed with a Wallac Microbeta scintillation counter (Perkin-Elmer, Waltham, MA, USA).

### Nitrate detection assay

MSCs (passage 4) were plated into 6-well plates, and when the cells were 80% confluent, 10 ng/mL IFN-γ and 10 ng/mL TNF-α were added for 12 h. Then, 1 mL of fresh serum-free conditioned medium was added to the plate. Conditioned medium was collected after 24 h. NO in conditioned medium was detected by Griess assay (S0021, Beyotime, China) according to the manufacturer’s protocol.

### Real-time PCR

MSCs (passage 4) were treated with 10 ng/mL IFN-γ and 10 ng/mL TNF-α for 10 h and 24 h. Total RNA was isolated with Trizol and reverse-transcribed into cDNA with a Bestar qPCR RT kit. mRNA expression of iNOS was detected by RT-PCR using Bestar real-time PCR master mix with an ABI Prism 7300 system. The primers used were as follows: iNOS, sense, GTTCTCAGCCCAACAATACAAGA, antisense, GTGGACGGGTCGATGTCAC; GA PDH, sense, AGGTCGGTGTGAACGGATTTG, antisense, TGTAGACCATGTAGTTGAGGTCA.

### Western blotting assay

MSCs (passage 4) were treated with 10 ng/mL IFN-γ and 10 ng/mL TNF-α for 2 h and 24 h. Total protein was acquired with RIPA buffer and quantified by BCA assay. The same amount of protein was analysed for expression of iNOS (ab49999, 1:1000, Abcam) and IκB-α (4812S, 1:1000, Cell Signaling Technology) by SDS-PAGE, and GAPDH was used as a control. The assays were performed according to previously described protocols [16].

### Cytokine detection

Serum cytokines were assayed by a Bio-Plex Pro Mouse Cytokine 23-plex Assay (M60009RDPD) using Luminex Technology according to the manufacturer’s protocol (Bio-Plex, Bio-Rad Laboratories).

### Immunofluorescence assay

MSCs (passage 4) were plated into 48-well plates, and when the cells had adhered to the plate, 10 ng/mL IFN-γ and 10 ng/mL TNF-α were added to the medium. After 2 h, the cells were fixed and blotted with 1% BSA at 37 °C for 30 min. Then, the cells were incubated with P65 (8242S, 1:200, Cell Signaling Technology) at 4 °C overnight. The cells were washed with PBS three times and then incubated with a fluorescent antibody (A11011, 1:200, Life Technologies) at 37 °C for 30 min. Finally, DAPI was used to stain the nuclei.

### Statistical analyses

All experiments were performed more than three times. Three technical replicates were performed in each experiment. Student’s *t* test was performed to analyse the differences between different groups. *P < 0.05 and **P < 0.01 represent significant differences.

## Results

### HNF-4α-MSCs promoted liver injury repair

**Fig. 1:**
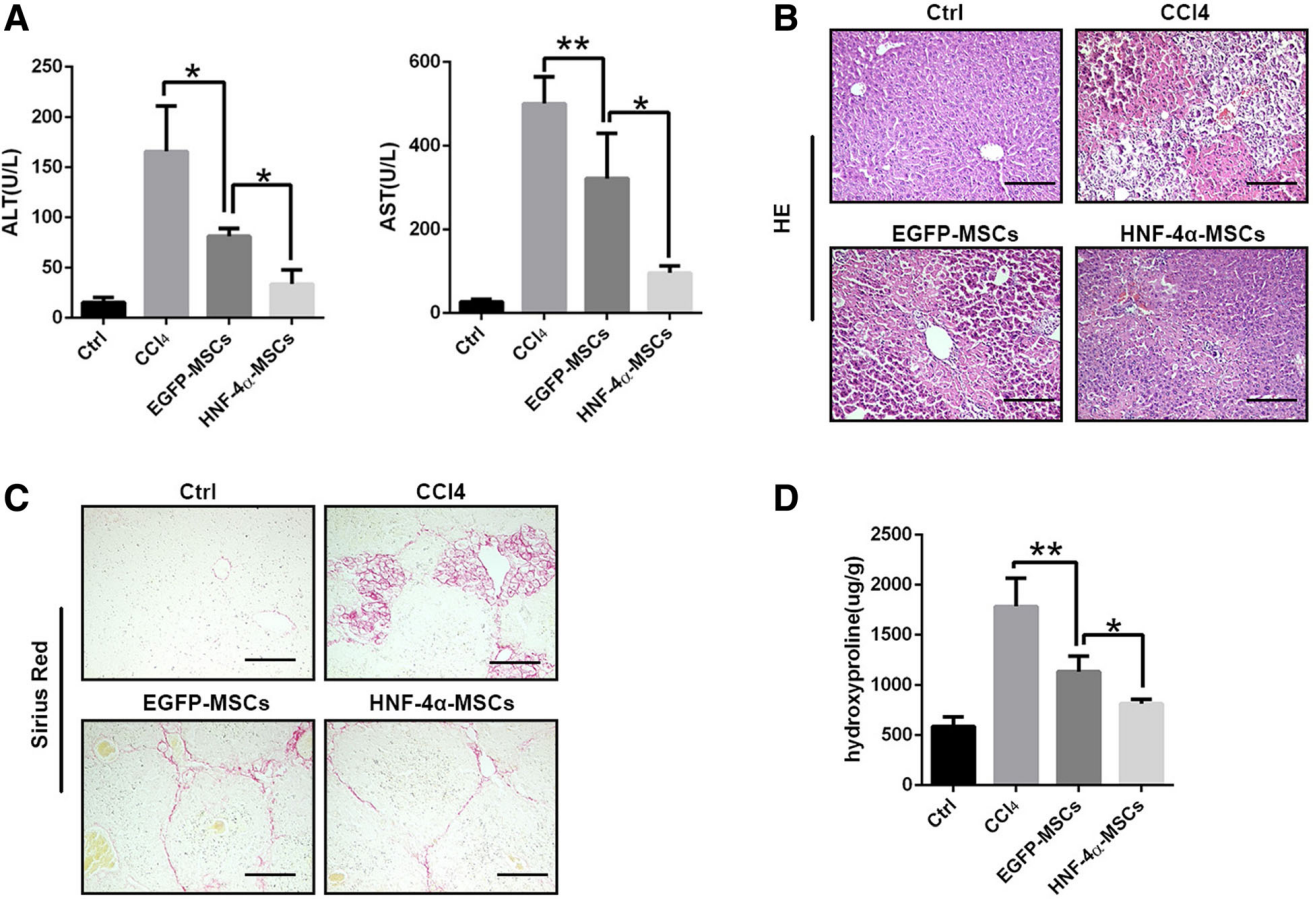
HNF-4α-MSCs promoted liver injury repair. EGFP-MSCs and HNF-4α-MSCs were injected into CCl_4_-induced mice. Mouse liver and serum samples were collected on the sixth week. Liver injury and fibrosis were detected. **a** ALT and AST levels were detected in serum. **b** HE staining was performed (× 200). **c** Sirius Red staining was performed (× 200). **d** Hydroxyproline was detected in mouse livers. (Five mice were used in each group. The data are represented as the mean ± S.D.) Scale bars, 100 μm

Mesenchymal stem cells (MSCs) were isolated from the bone marrow of 6–8-week-old mice. After passaging three times, MSCs were identified by their ability to differentiate into adipocytes and osteoblasts (Additional file 1: Figure S1). MSCs were transfected with adenovirus carrying HNF-4α (EGFP was used as a control). The efficiency of transfection was confirmed by fluorescent analysis and western blotting assay (Additional file 2: Figure S2). To determine the role of MSCs overexpressing HNF-4α in liver injury repair, we established a mouse liver injury model by using CCl_4_ administration and injected 1 × 10^6^ cells on the third week. We measured ALT and AST levels 3 weeks after cell administration. As shown in Fig. 1a, EGFP-MSCs reduced ALT and AST levels slightly, whereas HNF-4α-MSCs reduced ALT and AST levels to a greater extent. At the same time, we detected liver pathological alterations and liver fibrosis. As shown in Fig. 1b, c and d, EGFP-MSCs could reduce the hepatocyte necrosis and liver fibrosis induced by CCl_4_, and HNF-4α enhanced the effect of MSCs. In general, the above results suggested that EGFP-MSCs could alleviate liver injury and promote liver injury repair, while HNF-4α-MSCs enhanced the liver injury repair effect of MSCs.

### HNF-4α did not affect MSC homing

**Fig. 2:**
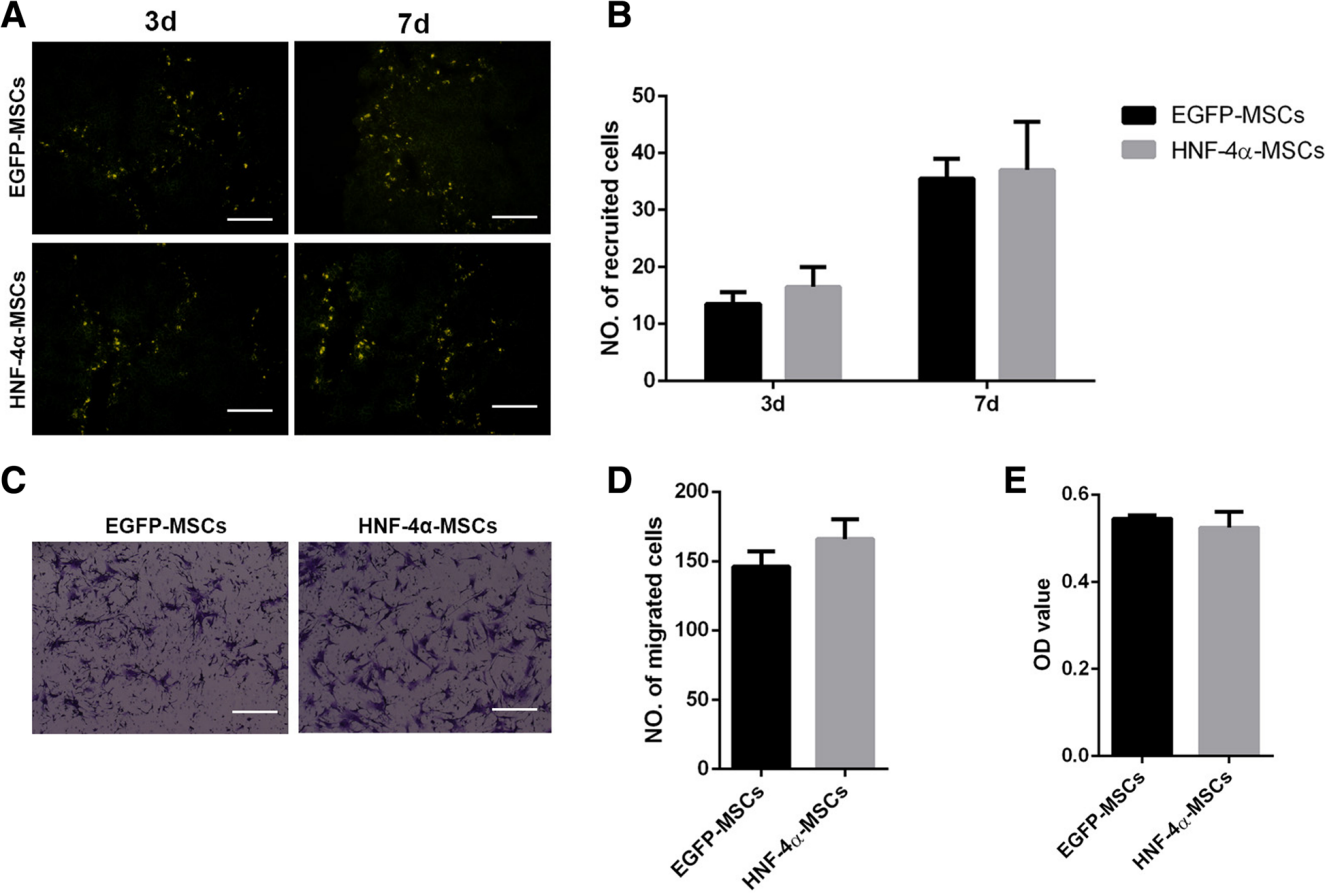
HNF-4α did not affect MSC homing. The migration and proliferation of MSCs were tested in vivo and in vitro*.* **a** Recruitment of MSCs was tested on the third day and seventh day after injection by fluorescent analysis (× 200). **b** Three fields of view were selected for each group to count the EGFP-positive cells. The mean of the three fields was used to represent the migrated cells for each group. **c** Transwell assays were performed to verify the migration of MSCs in vitro (× 200). Cell numbers were counted after 72 h. **d** Quantification of panel **c**. **e** A CCK-8 assay was performed to detect the proliferation of MSCs at 48 h. (Five mice were used in each group. The data are represented as the mean ± S.D.) Scale bars, 100 μm

As demonstrated before [17, 18], only when MSCs migrate to the injury site can their damage repair function be performed. Therefore, we detected the homing of MSCs to liver injury sites at different times. We tested MSC migration in vivo at 3 days and 7 days after their tail vein administration. Fluorescent observation of frozen sections of the liver showed that there was no difference between EGFP-MSC and HNF-4α-MSC recruitment at both 3 days and 7 days (Fig. 2a, b). We also detected the migration function of MSCs in vitro using a transwell assay. After 72 h, the migrated cells were counted. The results showed that there was no significant difference between the two groups (Fig. 2c, d). Furthermore, we tested the role of HNF-4α in the proliferation of MSCs by CCK-8 assay. MSCs, EGFP-MSCs and HNF-4α-MSCs were plated into 96-well plates, and 48 h later, a CCK8 assay was performed to detect cell viability. HNF-4α had no effect on the proliferation of MSCs (Fig. 2e).

### HNF-4α-MSCs inhibited Kupffer cell-associated inflammation

**Fig. 3:**
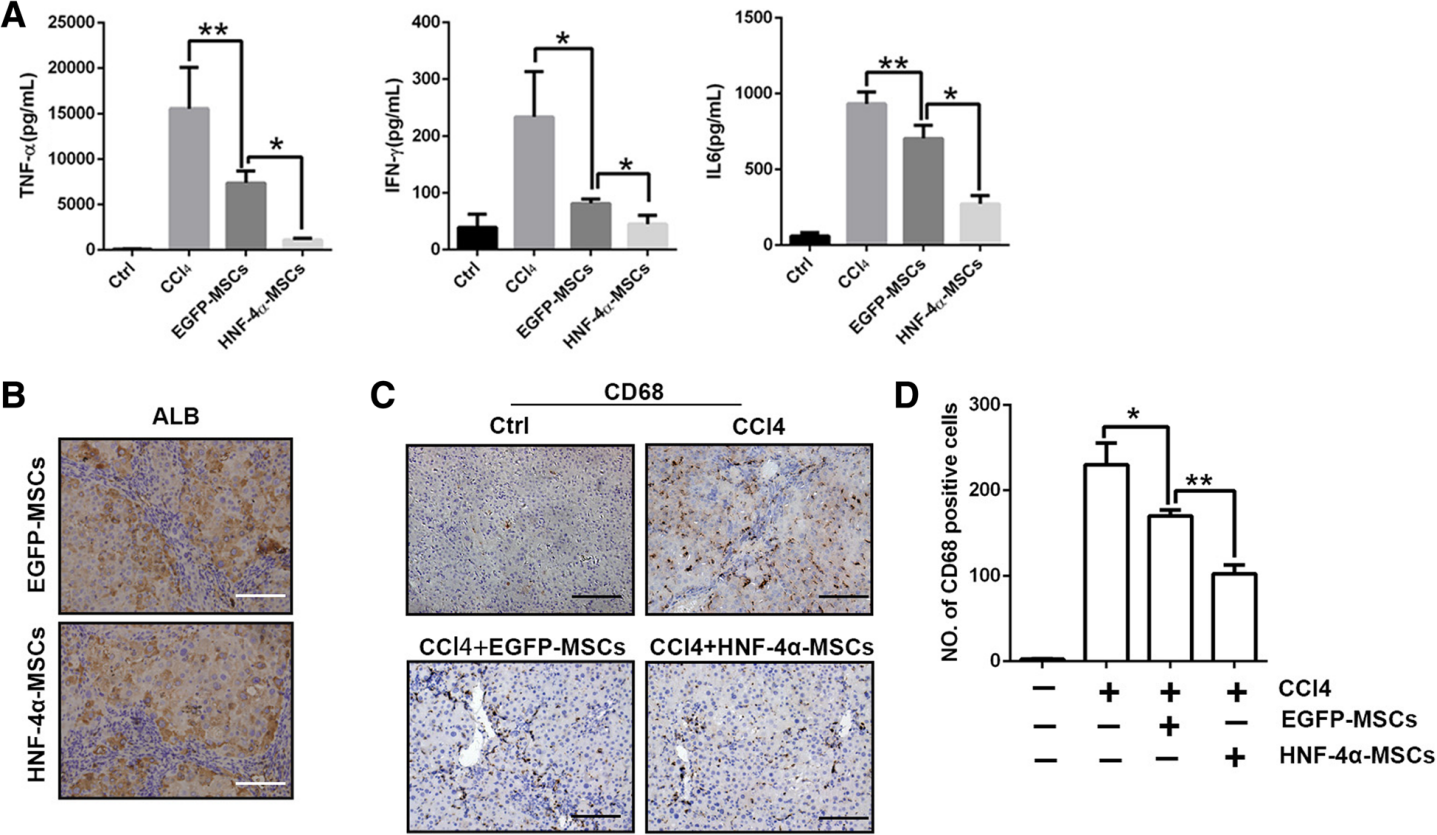
HNF-4α-MSCs inhibited Kupffer cell-associated inflammation. **a** Concentrations of the inflammatory factors TNF-α, IFN-γ and IL-6 were detected in mouse serum on the sixth week using a Bio-plex assay. **b** Expression of ALB was detected using IHC (× 320). **c** The Kupffer cell marker CD68 was detected in mouse livers using IHC (× 200). **d** Quantification of panel **c**. Three fields of view were selected for each group to count the CD68-positive cells. The mean of the three fields was used to represent the CD68^+^ cells for each group. (Five mice were used in each group. The data are represented as the mean ± S.D.) Scale bars, 100 μm

Inflammation is the main cause of liver injury, and MSCs were demonstrated to repair tissue injury by modulating inflammation [17, 19, 20]. We detected proinflammatory factors in mouse serum samples from different groups. As shown in Fig. 3a, HNF-4α-MSCs reduced the inflammatory factors in mouse serum samples considerably. As HNF-4α is a transcription activator that regulates hepatic differentiation, whether HNF-4α promotes hepatocyte differentiation in MSCs deserves consideration. First, we detected the expression of the hepatocyte marker ALB in the mouse livers. As shown in Fig. 3b, there were no differences in the expression of ALB, which suggested that hepatocyte differentiation did not reduce liver injury in the HNF-4α-MSC group. Kupffer cells are the main macrophage type in the liver sinusoids and contribute to host defence and inflammation accumulation [21]. CD68 immunohistochemistry staining showed that CCl_4_ induced Kupffer cell activation, and EGFP-MSCs inhibited the activation of Kupffer cells, which was enhanced by HNF-4α. In the HNF-4α-MSC group, Kupffer cell activation was reduced to a lower level (Fig. 3c, d). This is probably the reason why MSCs reduced liver inflammation.

### HNF-4α-MSCs exerted enhanced anti-inflammatory effects by increasing iNOS expression

**Fig. 4:**
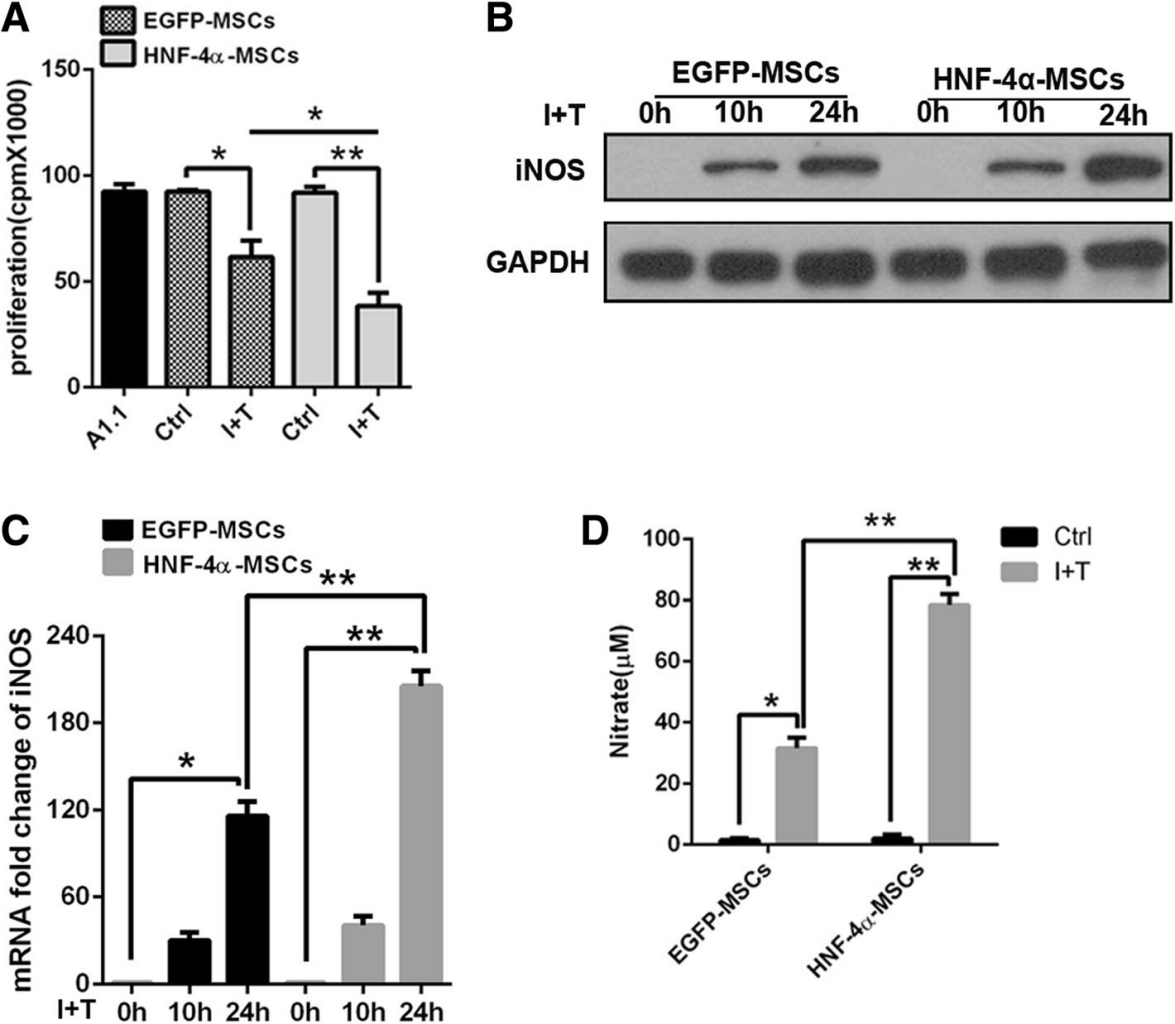
HNF-4α-MSCs exerted enhanced anti-inflammatory effects by increasing iNOS expression. **a** MSCs were treated with 10 ng/mL IFN-γ and 10 ng/mL TNF-α for 12 h and then co-cultured with A1.1 cells. After 24 h, the proliferation of A1.1 cells was detected using a ^3^H-Tdr assay. **b**, **c** MSCs were treated with 10 ng/mL IFN-γ and 10 ng/mL TNF-α for 10 h and 24 h. The expression of iNOS was detected using western blotting and RT-PCR assays. GAPDH was used as an internal control. **d** Conditioned medium from MSCs treated with 10 ng/mL IFN-γ and 10 ng/mL TNF-α was collected after 24 h. I+T, IFN-γ and TNF-α. Nitrate levels were detected (The data are represented as the mean ± S.D.)

To verify how MSCs inhibited Kupffer cell activation, we detected the anti-inflammatory effect of HNF-4α-MSCs in vitro by co-culture with lymphocyte A1.1 cells. As shown in previous studies, MSCs exert their anti-inflammatory function only after stimulation by inflammatory cytokines. MSCs stimulated with IFN-γ plus TNF-α (10 ng/mL) were co-cultured with A1.1 cells at a ratio of 1 MSCs:10 A1.1 cells. The ^3^H-Tdr assay results showed that HNF-4α-MSCs induced significantly more anti-inflammatory effects than EGFP-MSCs (Fig. 4a). Then, we detected iNOS expression in MSCs. Both EGFP-MSCs and HNF-4α-MSCs were treated with the inflammatory factors IFN-γ and TNF-α (10 ng/mL) for the indicated time. The expression of iNOS was higher in HNF-4α-MSCs than in control cells at 24 h. At the same time, we detected the concentration of nitrite in the conditioned medium of MSCs, which represents the NO content. NO is the key factor through which MSCs exert their anti-inflammatory effects [18]. After inflammatory factor stimulation, nitrate was increased significantly in the conditioned medium. Compared with EGFP-MSCs, HNF-4α-MSCs secreted more NO (Fig. 4d).

### HNF-4α promoted the immune regulation of MSCs through NF-κB signalling

**Fig. 5:**
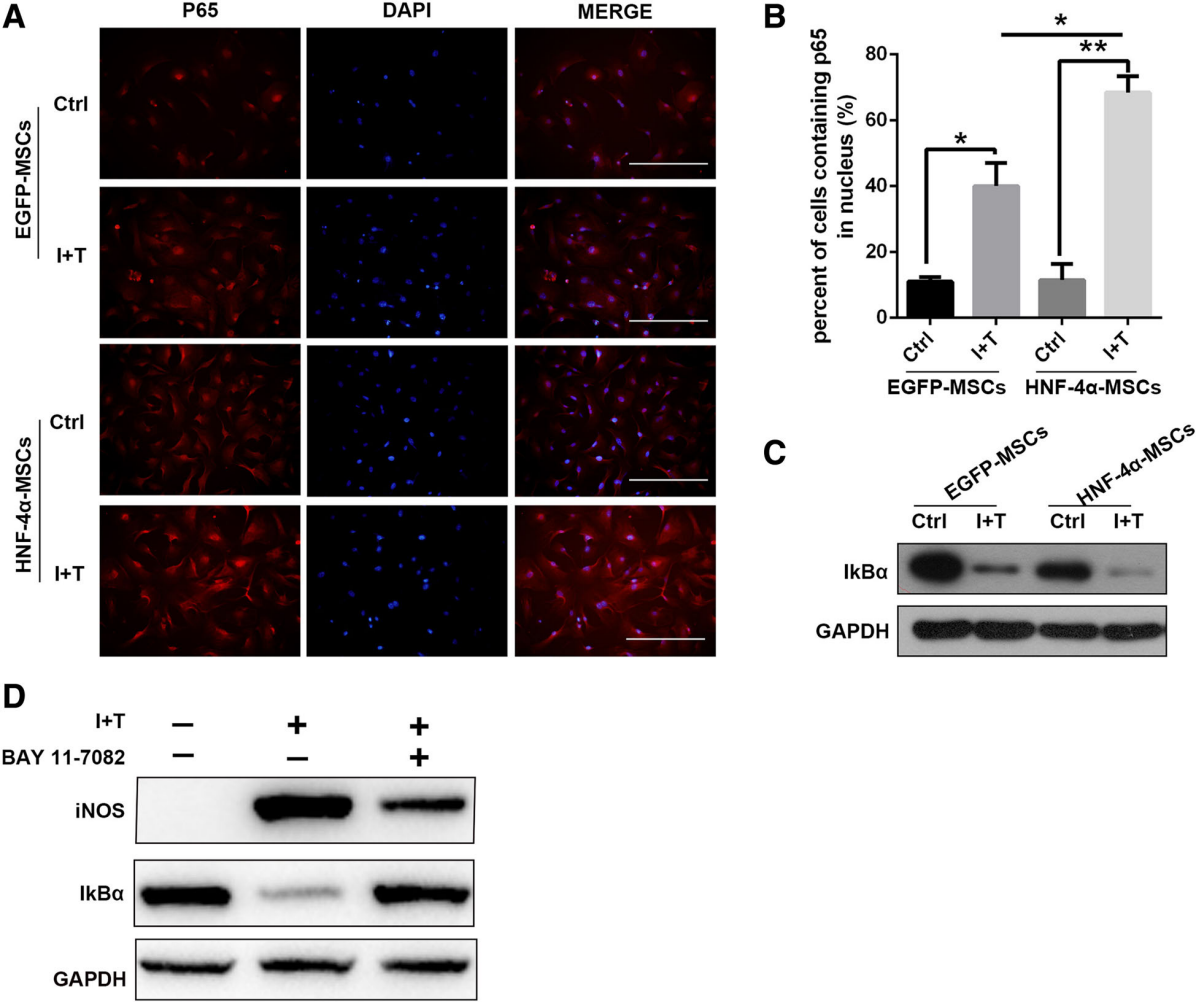
HNF-4α promoted the immune regulation of MSCs through NF-κB signalling. **a** P65 expression was detected in MSCs after TNF-α and IFN-γ treatment for 2 h by immunofluorescence staining. Nuclei were stained with DAPI (× 200). **b** The percentage of cells containing P65 in the nucleus was calculated. **c** Expression of IκB-α was detected by western blotting in MSCs after 10 ng/mL IFN-γ and 10 ng/mL TNF-α treatment for 2 h. GAPDH was used as an internal control. **d** Expression levels of IκB-α (treatment for 2 h) and iNOS (treatment for 24 h) were detected after NF-κB inhibitor BAY 11-7082 treatment in HNF-4α-MSCs. GAPDH was used as an internal control. I+T, IFN-γ and TNF-α. (The data are represented as the mean ± S.D.) Scale bars, 100 μm

The expression of iNOS can be regulated by several signalling pathways, including NF-κB, CCAAT enhancer binding protein beta (C/EBPβ), and signal transducer and activator of transcription 1 (STAT1) [22]. To investigate how HNF-4α overexpression regulates iNOS expression in MSCs, we first analysed the NF-κB signalling pathway. Interestingly, as shown in Fig. 5 a and b, the immunofluorescence staining results showed that after treatment with inflammatory factors for 2 h, more P65 was translocated into the nucleus in HNF-4α-MSCs. P65 translocation to the nucleus indicates NF-κB activation. In addition, we measured IκB-α expression after IFN-γ and TNF-α treatment for 2 h. Western blotting assays revealed that the expression of IκB-α was downregulated in HNF-4α-MSCs (Fig. 5c), which suggested NF-κB signalling pathway activation. In addition, we blocked the NF-κB signalling pathway using BAY 11-7082. We found that BAY 11-7082 treatment for 2 h effectively increased the level of IκB-α in HNF-4α-MSCs. In parallel, the expression of iNOS in HNF-4α-MSCs after IFN-γ and TNF-α treatment for 24 h was inhibited (Fig. 5d). As a result, HNF-4α upregulated the expression of iNOS via activation of the NF-κB signalling pathway.

## Discussion

Mesenchymal stem cells are a population of adult stem cells with self-renewal and multiple differentiation abilities. They can differentiate into mesodermal cells, such as bone, adipocytes, cartilage [23] and skeletal muscle [24]. MSCs can also differentiate into entodermal [25] and ectodermal [26] cells under special conditions. They have been demonstrated to be isolated from the bone marrow, umbilical cord, adipose tissue, liver [27], muscle [28], heart [29] and other tissues. MSCs can also be expanded easily in vitro. MSCs exhibit strong injury tropism and immunosuppression properties [30, 31]. Due to the characteristics described above, MSCs have great potential for use in clinical therapy.

Liver cirrhosis is an important health issue that commonly leads to liver failure and HCC. Currently, orthotopic liver transplantation is the most effective treatment, but this method is limited by donor shortages and immunological rejection. Cell therapy has become increasingly popular because of its efficiency and few side effects. Mesenchymal stem cells are the most popular stem cells for cell therapy. They can migrate to tissue injury sites and repair liver injury by differentiating into multiple types of cells and performing immunity regulation functions. However, not all of the effects of cell therapy are satisfactory.

Gene therapy is now another efficient method for treating some diseases. HNF-4α has been shown to be useful in treating HCC [14]. However, the safety concerns regarding virus vectors are disturbing. MSCs are a good vector of genes because of their low immunity and low tumourigenesis [6].

In our study, we found that HNF-4α overexpression facilitated the therapeutic effect of MSCs in liver cirrhosis. The liver function index, pathology structure and fibrosis indicated that HNF-4α accelerated the repair function of MSCs in liver injury. Inflammatory factor detection in mouse serum showed that inflammation was further reduced after HNF-4α-MSC transplantation and that macrophages were inhibited. In addition, enhanced repair function is not dependent on increased MSC migration, and HNF-4α does not affect MSC proliferation. Surprisingly, we found that there was no difference in ALB expression between the MSC and HNF-4α-MSC groups, which suggested that the hepatocyte differentiation of MSCs is not the key underlying mechanism. However, we found that HNF-4α-MSCs had enhanced immunosuppression ability.

NO is an important factor that kills immune cells [18]. Further study showed that HNF-4α promoted iNOS expression through the NF-κB pathway. In HNF-4α-MSCs, more p65 was relocated to the nucleus. When an NF-κB inhibitor was used, iNOS expression was reduced. The above data suggested that HNF-4α promoted the iNOS-dependent anti-inflammatory effect of MSCs by activating the NF-κB signalling pathway.

In conclusion, MSC modification by gene editing could enhance their therapeutic effect in liver cirrhosis. However, cell therapy combined with gene therapy needs further study.

## Conclusions

In this study, we found that MSCs overexpressing HNF-4α exerted better therapeutic effects than control MSCs against mouse liver cirrhosis by enhancing their NF-κB-dependent anti-inflammatory effect. Thus, gene modification is likely a promising method for improving the effects of cell therapy.

## Additional files

Additional file 1: **Figure S1.** Identification of MSCs according to their differentiation potential. MSCs were isolated from mouse bone marrow and identified after passaging three times. MSCs were induced to differentiate into adipocytes and osteoblasts with differentiation medium for 7 days and 21 days, respectively, and then stained with oil red and alizarin red. (JPG 697 kb)

Additional file 2: **Figure S2.** Confirmation of adenovirus transfection. MSCs were transfected with adenoviruses carrying EGFP and EGFP-HNF-4α. (A) Fluorescence was observed under a fluorescence microscope (× 200). (B) HNF-4α protein levels were detected by western blotting. (JPG 246 kb)

## Abbreviations

AV: Adenovirus
C/EBPβ: CCAAT enhancer binding protein beta
CCl_4_: Carbon tetrachloride
EGFP: Enhanced green fluorescence protein
HCC: Hepatocellular carcinoma
HNF-4α: Hepatocyte nuclear factor-4α
iNOS: Inducible nitric oxide synthase
MSCs: Mesenchymal stem cells
NF-κB: Nuclear factor kappa B
OLT: Orthotopic liver transplantation
STAT1: Signal transducer and activator of transcription 1
