# Supplementary figures and images for: Mesenchymal stem cells overexpressing hepatocyte nuclear factor-4 alpha alleviate liver injury by modulating anti-inflammatory functions in mice
Source: Stem Cell Res Ther. 2019 May 27;10:149. doi: 10.1186/s13287-019-1260-7 (PMC6537220; doi:10.1186/s13287-019-1260-7)

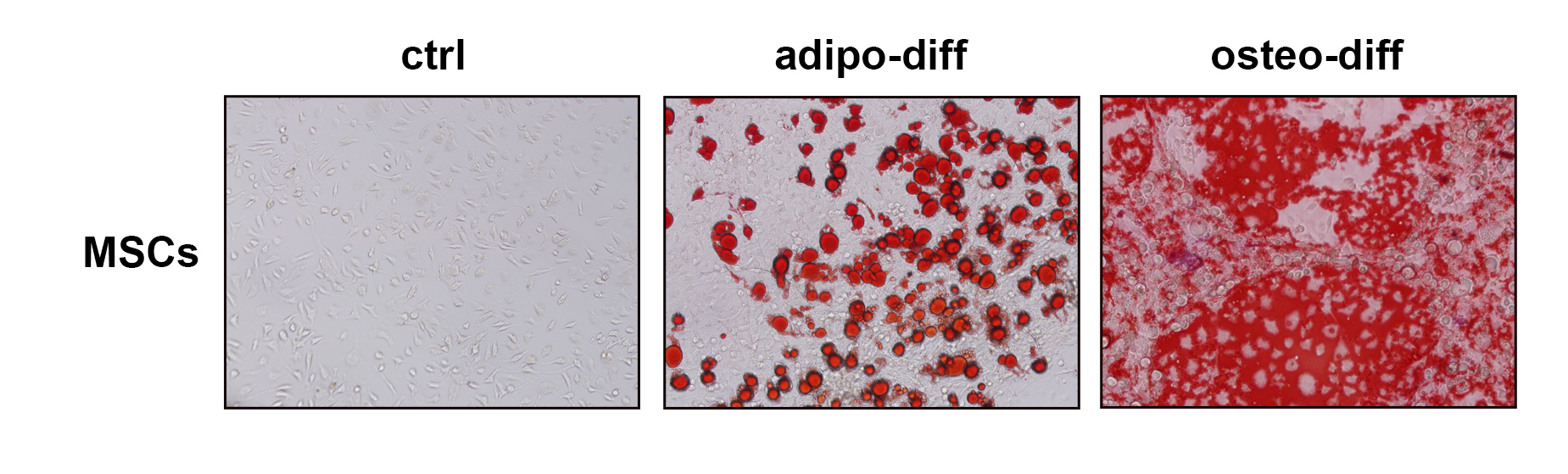

Supplement: Supplementary file 1 — Figure S1. Identification of MSCs according to their differentiation potential. MSCs were isolated from mouse bone marrow and identified after passaging three times. MSCs were induced to differentiate into adipocytes and osteoblasts with differentiation medium for 7 days and 21 days, respectively, and then stained with oil red and alizarin red. (JPG 697 kb) [file 13287_2019_1260_MOESM1_ESM.jpg]

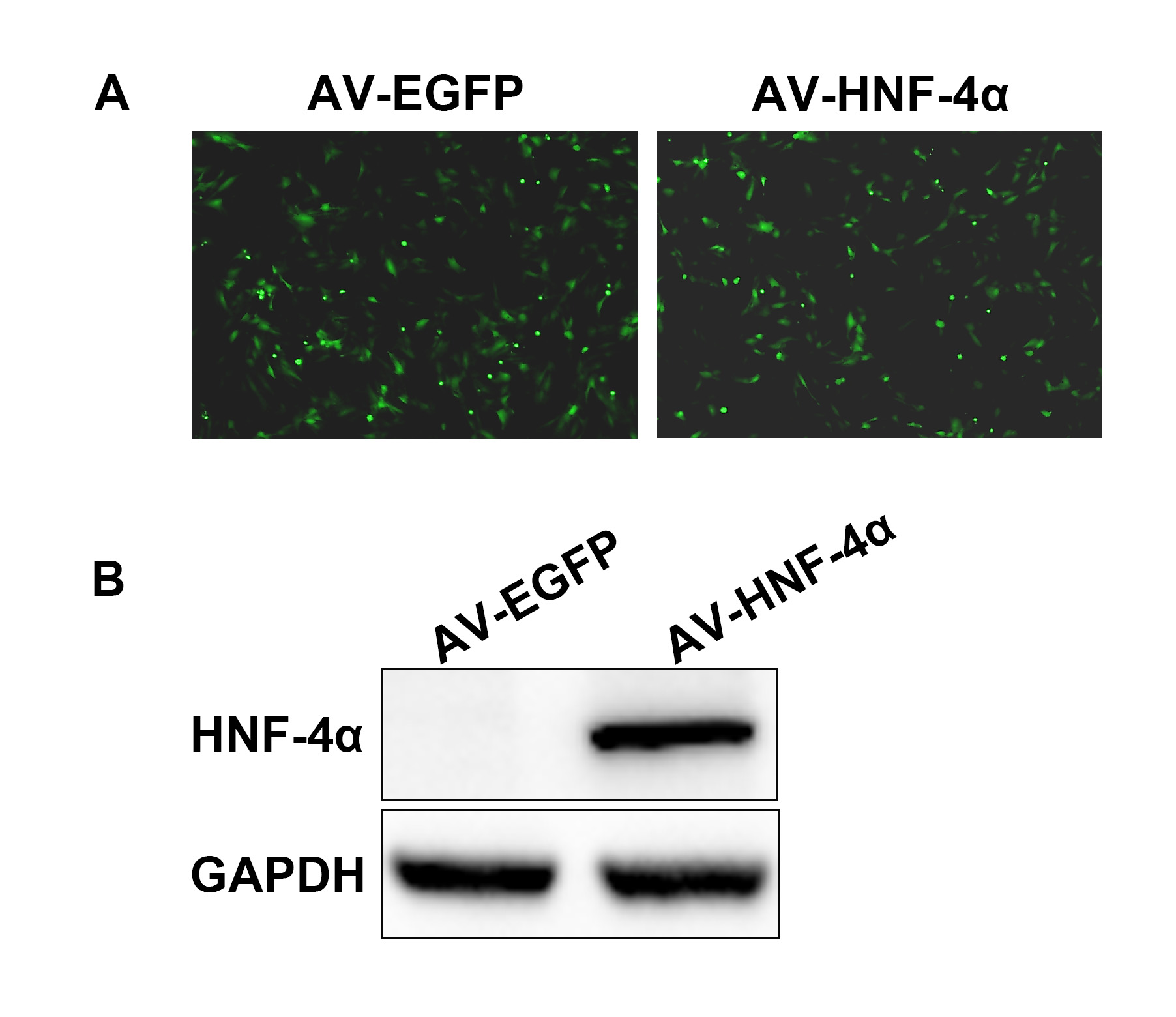

Supplement: Supplementary file 2 — Figure S2. Confirmation of adenovirus transfection. MSCs were transfected with adenoviruses carrying EGFP and EGFP-HNF-4α. (A) Fluorescence was observed under a fluorescence microscope (× 200). (B) HNF-4α protein levels were detected by western blotting. (JPG 246 kb) [file 13287_2019_1260_MOESM2_ESM.jpg]
